# NK Cell Reconstitution in Paediatric Leukemic Patients after T-Cell-Depleted HLA-Haploidentical Haematopoietic Stem Cell Transplantation Followed by the Reinfusion of iCasp9-Modified Donor T Cells

**DOI:** 10.3390/jcm8111904

**Published:** 2019-11-07

**Authors:** Helena Stabile, Paolo Nisti, Cinzia Fionda, Daria Pagliara, Stefania Gaspari, Franco Locatelli, Angela Santoni, Angela Gismondi

**Affiliations:** 1 Department of Molecular Medicine, Istituto Pasteur-Fondazione Cenci Bolognetti, Sapienza University of Rome, Laboratory affiliated to Istituto Pasteur Italia-Fondazione Cenci Bolognetti, 00161 Rome, Italy; paolo.nisti@yahoo.it (P.N.); cinzia.fionda@uniroma1.it (C.F.); angela.santoni@uniroma1.it (A.S.); 2 Department of Pediatric Hematology/Oncology, Istituto di Ricovero e Cura a Carattere Scientifico (IRCCS) Ospedale Pediatrico Bambino Gesù, 00165 Rome, Italy; daria.pagliara@opbg.net (D.P.); stefania.gaspari@opbg.net (S.G.); franco.locatelli@opbg.net (F.L.); 3 Department of Pediatrics, Sapienza, University of Rome, 00161 Rome, Italy

**Keywords:** NK cell subsets, Bone marrow transplantation, haematological disease, iCasp-9 HSCT

## Abstract

T-cell-depleted (TCD) human leukocyte antigen (HLA) haploidentical (haplo) hematopoietic stem cell transplantation (HSCT) (TCD-haplo-HSCT) has had a huge impact on the treatment of many haematological diseases. The adoptive transfer of a titrated number of T cells genetically modified with a gene suicide can improve immune reconstitution and represents an interesting strategy to enhance the success of haplo-HSCT. Natural killer (NK) cells are the first donor-derived lymphocyte population to reconstitute following transplantation, and play a pivotal role in mediating graft-versus-leukaemia (GvL). We recently described a CD56^low^CD16^low^ NK cell subset that mediates both cytotoxic activity and cytokine production. Given the multifunctional properties of this subset, we studied its functional recovery in a cohort of children given α/βT-cell-depleted haplo-HSCT followed by the infusion of a titrated number of iCasp-9-modified T cells (iCasp-9 HSCT). The data obtained indicate that multifunctional CD56^low^CD16^low^ NK cell frequency is similar to that of healthy donors (HD) at all time points analysed, showing enrichment in the bone marrow (BM). Interestingly, with regard to functional acquisition, we identified two groups of patients, namely those whose NK cells did (responder) or did not (non responder) degranulate or produce cytokines. Moreover, in patients analysed for both functions, we observed that the acquisition of degranulation capacity was not associated with the ability to produce interferon-gamma (IFN-γ Intriguingly, we found a higher BM and peripheral blood (PB) frequency of iCas9 donor T cells only in patients characterized by the ability of CD56^low^CD16^low^ NK cells to degranulate. Collectively, these findings suggest that donor iCasp9-T lymphocytes do not have a significant influence on NK cell reconstitution, even if they may positively affect the acquisition of target-induced degranulation of CD56^low^CD16^low^ NK cells in the T-cell-depleted haplo-HSC transplanted patients.

## 1. Introduction

Allogeneic hematopoietic stem cell transplantation (HSCT) plays a relevant role in the treatment of many haematological diseases, both malignant and nonmalignant, representing a life-saving procedure [1]. In particular, HLA-haploidentical HSCT (haplo-HSCT), where the donor and recipient share one HLA haplotype while the other is fully mismatched, represents a valuable option for those patients in need of an allograft who lack an HLA-compatible donor; however, in order to prevent the occurrence of severe graft-versus-host disease (GvHD), a deep T cell depletion of the graft remains fundamental [2,3]. 

The Achilles heel of T-cell-depleted HLA haplo-HSCT is related to the delay in the reconstitution of adaptive immunity that exposes patients to opportunistic infections leading to treatment failure [4]. Great efforts have been made to boost post-transplant immune recovery and augment the graft-versus-leukaemia (GvL) effect. Several groups have shown that the adoptive transfer of a controlled number of donor-derived T cells can improve immune reconstitution, representing an interesting tool to enhance the success of haplo-HSCT, although it is not without risks [5,6,7,8]. Donor T cells, administrated simultaneously or post-HSCT, could cause adverse effects, including cytokine release syndrome and GvHD, leading to treatment failure [9]. In order to increase the feasibility of donor T cell adoptive transfer, a safety mechanism to eliminate the infused cells in case of side effects has been developed. In this regard, the transduction of adoptive transfer cells with a safety switch allows us to selectively eliminate only the cells responsible for the unwanted toxicity. One of the most promising suicide genes is represented by the inducible caspase-9 (iCasp9), associated with a ΔCD19 marker to selectively follow and isolate the transduced T cells [10,11]. In vivo persistence of these safety-switch-modified T cells up to two years post-infusion has been reported, together with a faster recovery of endogenous T cells [12].

Natural killer (NK) cells are the first donor-derived lymphocyte population to reconstitute following transplantation, and play a pivotal role in mediating the GvL effect by the production of inflammatory cytokines and by direct lysis of leukemic blasts [13,14]. In addition to their anticancer effect, NK cells are shown to become activated and proliferate in response to viral infections, like cytomegalovirus (CMV), which is one of the main obstacles during the first month post-HSCT [15,16,17].

Natural killer cells are a heterogeneous population and distinct NK cell subsets can be identified according to the cell surface receptor profile and functional ability [18,19]. In recent years, we have focused our efforts on a multifunctional NK cell subset characterized by a low expression level of CD56 (neuronal adhesion molecule NCAM) and CD16 (low-affinity Fc-receptor IIIA) [20]. We demonstrated that CD56^low^CD16^low^ NK cells are high IFN-γ producers and are able to kill acute leukaemia cells. Moreover, we found that, despite the enrichment of CD56^low^CD16^low^ NK cells in both the bone marrow (BM) and peripheral blood (PB) of children with acute lymphoblastic leukaemia (ALL), with respect to healthy donors (HD), their functionality was impaired. Recently, we also described a key role for this NK cell subset in multiple myeloma (MM), as CD56^low^CD16^low^ NK cells are able to recognize and kill MM cells but, similarly to ALL patients, their function is impaired in MM patients [21]. 

NK cell functional characteristics are deeply influenced by the type of transplantation, especially by the HSC cell source and by the manipulation of the graft. In particular, conflicting results have been reported concerning the role played by graft T cell content on NK cell development and functional maturation [22]. 

Here, we analysed NK cell reconstitution in a cohort of paediatric leukemic patients undergoing haploidentical α/β T-cell-depleted HSCT followed by donor iCasp9-modified T cell infusion, focusing on CD56^low^CD16^low^ NK cell functional recovery. 

## 2. Methods

### 2.1. Cell Source

PB and BM cells were obtained from 35 leukemic patients after T-cell-depleted hematopoietic stem cell transplantation plus reinfusion of iCasp9-modified donor T cells at Bambino Gesù Children’s Hospital, Rome, Italy (Table S1). PB and BM samples from some patients were analysed at different time points after transplantation, so a total of 56 BM and 52 PB samples were analysed. The study was approved by the institutional ethics committees and informed consent was obtained from donors, patients and/or their legal guardians. 

### 2.2. Multicolour Immunofluorescence and Flow Cytometry Analysis

Freshly isolated PB and BM mononuclear cells were stained using the appropriate antibody (Ab) combination (see Table S2) and subjected to flow cytometry analysis. Intracellular staining with appropriate mAb was performed after fixation and permeabilization with a BD cytofix/cytoperm (Biosciences, San Jose, CA, USA) flow cytometer, and flow cytometry analysis was performed with FlowJo 9.2.3 (TreeStar, Ashland, OR, USA).

### 2.3. Degranulation Assay and IFN-γ Production 

NK cells from PB or BM were co-cultured with MHC class I negative human erythroleukemia cell line K562 at 1:1 effector/target (E/T) ratio for 3 h, in the presence of 50 M monensin (BD Biosciences) for the last 2 h; degranulation was assessed by evaluating the CD107a expression in NK cell subsets.

To evaluate intracellular IFN-γ production, mononuclear cells were incubated with IL-12 (25 ng/mL) plus IL-15 (50 ng/mL) (PeproTech, London, UK) at 37 °C. After 1 h, 5 g/mL brefeldin A were added, and cells were incubated for an additional 6 h. Cells were subsequently fixed, permeabilized, stained with anti-IFN-γ-APC and analysed by flow cytometry. 

### 2.4. Statistical Analysis 

ANOVA or *t*-tests were used to compare independent groups. Statistical analyses were performed using PRISM 6.0 (GraphPad, La Jolla, CA, USA).

## 3. Results and Discussion

### 3.1. Impact of iCasp9-Modified Donor T Cells on NK Cell Recovery after HSCT

We monitored a cohort of 35 children who received T-cell-depleted haploidentical HSCT plus reinfusion of iCasp9-modified donor T cells (Table S1) [22]. According to this protocol, patients undergoing T-cell-depleted HSCT on day 14 ± 4 after transplantation were infused with a single intravenous dose of haploidentical iCasp9-T cells (0.25 × 10^6^, 1 × 10^6^ or 4 × 10^6^ cells/kg of body weight; see Table S1). 

### 3.2. NK Cell Distribution in BM and PB of iCasp9-HSCT Patients

**Figure 1:**
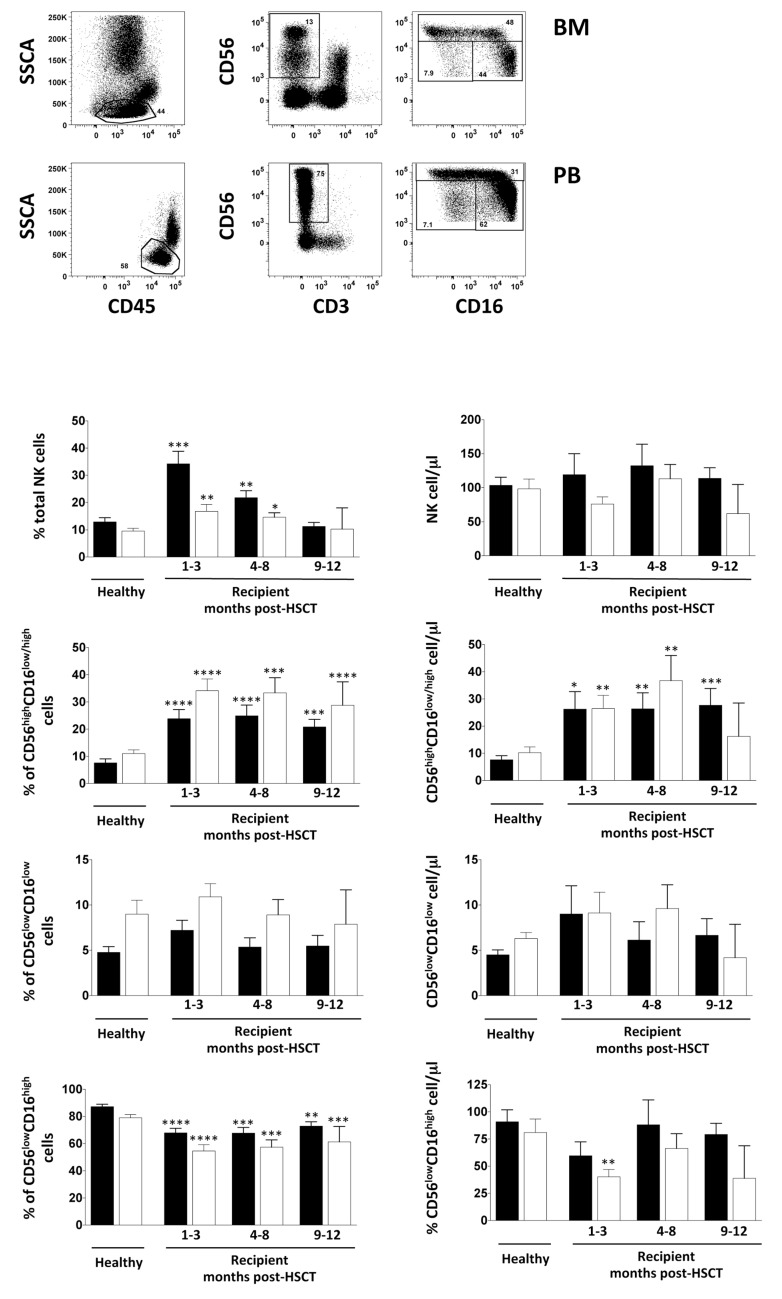
BM and PB CD56^high^CD16^+/−^, CD56^low^CD16^low^ and CD56^low^CD16^high^ NK cell subset recovery after T-cell-depleted HSCT followed by iCas9 donor T cell infusion. Cells freshly isolated by density gradient centrifugation from BM and PB of paediatric healthy donors and iCas9 haplo-HSCT recipients were analysed by flow cytometry (gating strategy in upper panel). Bar graphs represent the percentage and absolute cell number/μL of total NK cells among lymphocytes and of CD56^high^CD16^+/−^, CD56^low^CD16^low^ and CD56^low^CD16^high^ NK cell subsets gated on CD56^+^CD3^−^ NK cells in BM (white) and PB (black). Error bars represent SEM. Unpaired *t*-test, * *p* < 0.05; ** *p* < 0.005; *** *p* < 0.0002; **** *p* < 0.0001. Healthy: BM *n* = 16, PB *n* = 14; Patients: BM *n* = 56, PB *n* = 52.

First, we addressed a possible effect of iCasp9-modified donor T cells on early NK cell reconstitution by monitoring the NK cell frequency and number in both BM and PB for one year by comparing them with age-matched HD. In line with previous observations, we found a significant increase of NK cell frequency in both PB and BM in the first eight months after transplantation, with a higher NK cell percentage in PB with respect to BM. However, no difference was found in terms of the absolute number of NK cells between patients and HD (Figure 1). To gain insight into the possible selective effect of iCasp9-modified donor T cells on the reconstitution of different NK cell subsets by polychromatic flow cytometry, we further classified the NK cells into three subsets according to their CD56 and CD16 surface expression: CD56^high^CD16^+/−^, CD56^low^CD16^low^ and CD56^low^CD16^high^ NK cells. Consistent with our previous results for T-cell-depleted HLA-haploidentical HSC transplanted patients and data published by other groups on different types of transplant setting, the distribution of NK cell subpopulations post-HSCT is remarkably different from that of HD, showing a significantly higher frequency and absolute number of CD56^high^CD16^+/−^ NK cells in the recipients, counterbalanced by a low frequency of CD56^low^CD16^high^ NK cells both in PB and BM up to one year after transplantation [23,24,25]. By contrast, multifunctional CD56^low^CD16^low^ NK cell frequency and their absolute number are similar to the reference range of HD at all time points analysed, showing enrichment in the BM (Figure 1). Collectively, these findings suggest that donor iCasp9-T lymphocytes do not have a significant influence on NK cell and NK cell subset reconstitution in T-cell-depleted HLA-haploidentical HSC transplanted patients. Similar results were described for other transplant settings in which the overall percentage of NK cells was not influenced by the T cell graft content, whereas the latter was reported to have an impact on NK cell functional recovery [24,26,27]. On the contrary, it has been previously reported that iCasp9-T cells promote the reconstitution of endogenous naïve T lymphocytes, which is usually delayed up to one year after HSCT thanks to their long persistence in the recipients [12]. 

### 3.3. NK Cell Effector Functions in iCasp9-HSCT Patients

To investigate whether iCasp9 T cells could play a role in NK cell functional maturation, we evaluated the functional capability of NK cells isolated from BM and PB of iCasp9-HSCT patients throughout the one-year follow-up. 

To assess NK cell degranulation ability, we evaluated the proportion of CD107a positive CD56^high^CD16^+/−^, CD56^low^CD16^low^ and CD56^low^CD16^high^ NK cells once co-cultured with HLA class-I deficient K562 target cells. The cytokine production was measured by estimating the capacity of the three NK cell subsets to produce IFN-γ upon IL-12 plus IL-15 stimulation in an intracellular assay. 

**Figure 2:**
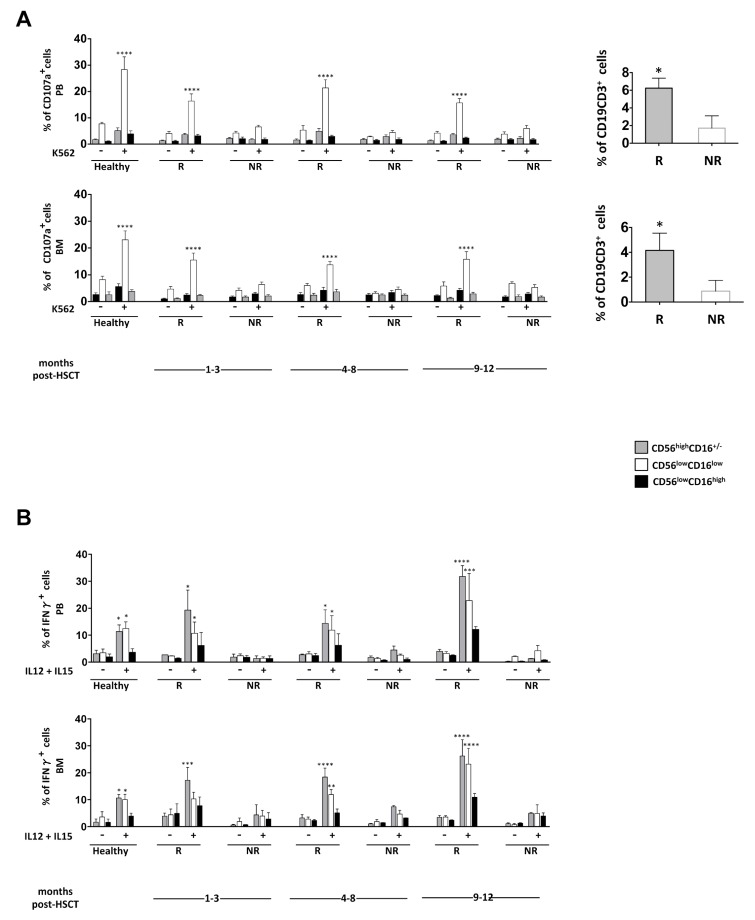
Impact of iCas9 donor T cells on BM and PB CD56^low^CD16^low^ NK cell effector functions at different time points after T-cell-depleted HSCT. (**A**) Cells freshly isolated from BM or PB of paediatric transplanted patients and healthy donors were co-cultured for 3 h with K562 target cells at 1:1 effector/target cells ratio and the degranulation ability of CD56^high^CD16^+/−^(grey), CD56^low^CD16^low^ (white) and CD56^low^CD16^high^ (black) NK cell subsets gated on CD56^+^CD3^−^ NK cells was assessed by measuring the percentage of CD107a positive cells by flow cytometry. CD107a-positive cells of two groups of patients are shown: R, responder and NR, nonresponder. The bar graphs represent the mean value ± SEM of the percentage of positive cells in BM and PB analysed at different time points after haplo-HSCT. ANOVA test, **** *p* < 0.0001. (Left panel). Healthy: PB *n* = 6, BM *n* = 5; R: PB *n* = 26, BM *n* = 19; NR: PB *n* = 18, BM *n* = 22. The percentage of iCas9 donor T cells gated on CD3^+^ T cells of responder (R) (grey) and nonresponder (NR) (white) patients for degranulation is shown. The bar graphs represent the mean value ± SEM (right panel). Unpaired *t*-test, * *p* < 0.05. (**B**) Freshly isolated cells from BM or PB of paediatric transplanted patients and healthy donors were stimulated overnight with IL-12 (25 ng/mL) plus IL-15 (50 ng/mL). The percentage of IFN-γ-positive cells into CD56^high^CD16^+/−^ (grey), CD56^low^CD16^low^ (white) and CD56^low^CD16^high^ (black) NK cell subsets gated on CD56^+^CD3^−^ NK cells was assessed by flow cytometry analysis. The bar graphs represent the mean value ± SEM of the percentage of positive cells analysed. ANOVA test, * *p* < 0.05; ** *p* < 0.005; *** *p* < 0.0002; **** *p* < 0.0001. IFN-γ positive cells of two groups of patients are shown: R, responder and NR, nonresponder. Healthy: PB *n* = 5, BM *n* = 5; R: PB *n* = 6, BM *n* = 7; NR: PB *n* = 9, BM *n* = 9.

**Figure 3:**
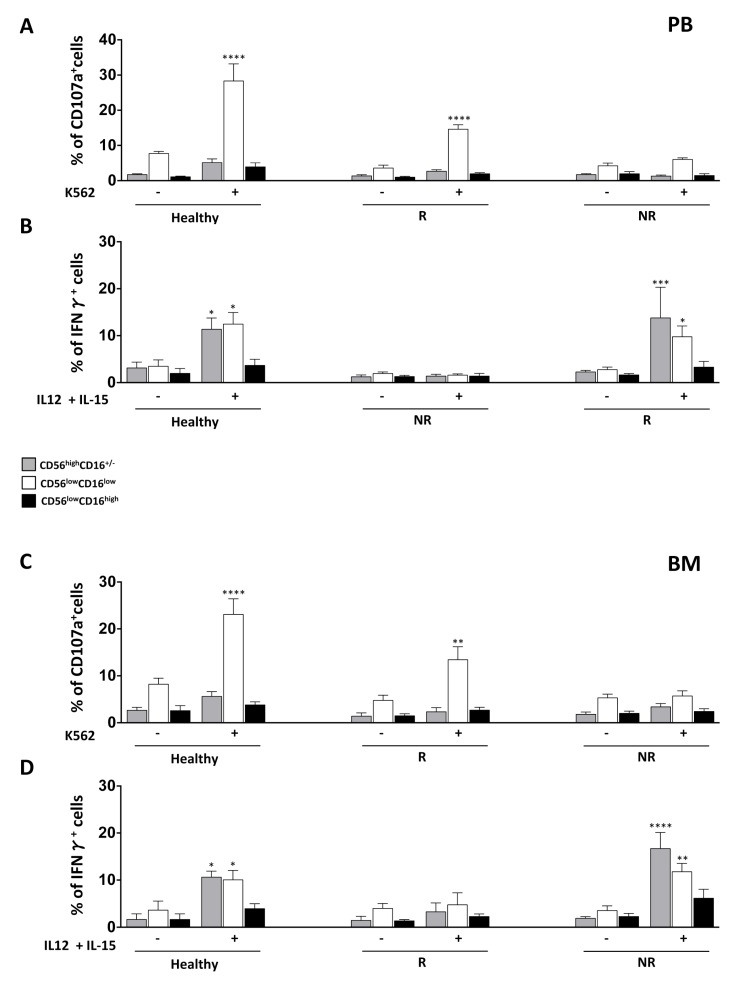
Dissociation between the recovery of degranulation ability and IFN-γ production by BM and PB CD56^low^CD16^low^ NK cells in patients given T-cell-depleted HSCT followed by iCas9 donor T cell infusions. The degranulation ability and IFN-γ production by CD56^high^CD16^+/−^(grey), CD56^low^CD16^low^ (white) and CD56^low^CD16^high^ (black) NK cell subsets gated on CD56^+^CD3^−^ NK cells were assessed as described in Figure 2. The bar graphs represent the mean value ± SEM of the percentage of positive cells analysed. ANOVA test, * *p* < 0.05; ** *p* < 0.005; *** *p* < 0.0002; **** *p* < 0.0001. R: PB *n* = 8, BM *n* = 3; NR: PB *n* = 5, BM *n* = 6.

Interestingly, the patients analysed can be divided into two distinct groups, according to their different functional abilities. The analysis of degranulation upon target cell stimulation or IFN-γ production highlights patients endowed with (responder) or without (nonresponder) functional competence (Figure 2, panels A and B; the main patient characteristics are reported in Table S3). We indicated as responder and nonresponder patients endowed with a degranulating capacity and IFN-γ production higher or lower than 10%, respectively. In particular, with respect to degranulation ability, as previously reported, only the CD56^low^CD16^low^ NK cell subset was able to degranulate at one month post-T-cell-depleted HSCT, although to a lesser extent than HD. By contrast, the ability to produce IFN-γ was found in both CD56^low^CD16^low^ and CD56^high^CD16^+/−^ NK cell subsets at levels comparable to those in HD. Intriguingly, in patients analysed for both functions, we observed that the acquisition of degranulation ability was not correlated with the ability to produce IFN-γ (Figure 3 and Figure S2; the main patient characteristics are reported in Table S4). In spite of the different functional ability shown by CD56^low^CD16^low^ and CD56^high^CD16^+/−^ NK cells from these two groups of patients, we found no major difference in the surface phenotype (i.e., expression of activating and inhibitory receptors, as well as of cytokine receptors) of these cell subsets between the two groups (Figure S1). Moreover, neither the type of leukaemia (ALL vs. AML) nor the occurrence of viral infections or of GVHD after the allograft significantly affected the CD56^low^CD16^low^ NK cell functional recovery.

Intriguingly, when we investigated the persistence of iCas9 donor T cells (i.e., CD3^+^CD19^+^ T cells) in the two groups of patients, we observed a higher BM and PB frequency of iCas9 donor T cells at three months post-transplantation only in the transplanted patients characterized by the presence of CD56^low^CD16^low^ NK cells able to degranulate (Figure 2, right panel).

A dissociation between the recovery of cytokine production and cytolytic function following allo-HSCT has been reported previously; the ability to kill is usually recovered before the capacity to produce IFN-γ upon target cell stimulation [28,29,30]. Moreover, it was described that T cell presence, either in the HSCT graft content or as adoptive immunotherapy following transplantation, could enhance NK cell functional maturation. NK cells collected from T-cell-depleted transplant recipients not receiving immunosuppressive therapy display impaired target-induced degranulation and IFN-γ production, whereas degranulation is normal and recovered earlier with respect to IFN-γ production in patients undergoing T-cell-replete transplants and receiving immunosuppression, suggesting a role for T cells in NK cell education. In sharp contrast, the recovery of IFN-γ production following cytokine, but not target stimulation, characterizes more immature NK cell populations [30].

We previously described a significant delay in the recovery of CD56^low^CD16^low^ NK cell degranulation capacity, while both the CD56^low^CD16^low^ and CD56^high^CD16^+/−^ NK cell subsets quickly acquired cytokine-induced IFN-γ production in T-cell-depleted HLA-haploidentical HSC transplanted patients [23]. In this new cohort of patients receiving iCas9 donor T cell infusions, we observed a faster recovery of the CD56^low^CD16^low^ NK cell subset target-induced degranulation, although this was restricted to the group of patients who had a greater percentage of iCasp9 donor T cells in PB. These findings are in line with the data reported in the literature and suggest an impact of donor-derived T cells on CD56^low^CD16^low^ NK cell functional recovery. However, the mechanisms explaining the faster functional recovery of NK cells in these transplanted patients require further investigation. 

## 4. Conclusions

Herein we suggest a positive influence of iCas9 donor-derived T cells in the earlier recovery of target-induced degranulation of CD56^low^CD16^low^ NK cells in paediatric leukemic patients. We propose that the presence of donor-derived T cells may promote NK cell functional recovery. Indeed, the cytokines produced by these T cells may positively affect NK cell maturation, leading to the acquisition of degranulating capacity (Figure S3). The quick recovery of CD56^low^CD16^low^ NK cell subset functionality should be considered an interesting result of this new hematopoietic transplantation setting; however, further research is needed to achieve fully functional competent NK cells (cytotoxicity and cytokine production) in order to take advantage of both NK cell-mediated antileukemic and antiviral immunity in transplanted patients. 

## Supplementary Materials

The following are available online at https://www.mdpi.com/2077-0383/8/11/1904/s1, Figure S1: Receptor phenotype of BM and PB NK cell subsets from CD107a and IFN-γ responder and non responder patients; Figure S2: Recovery of degranulation ability by BM and PB CD56^low^CD16^low^ NK cells in patients given T-cell depleted HSCT followed by iCas9 donor T cell infusion. Figure S3: Schematic representation of CD56^low^CD16^low^ NK cell functional recovery and its correlation with the frequence of CD19^+^CD3^+^ cells; Table S1: Patient characteristics; Table S2: characteristics of patients used in Figure 2; Table S3: characteristics of patients used in Figure 3; Table S4: characteristics of patients used in Figure 3.
