# Supplementary material for: NK Cell Reconstitution in Paediatric Leukemic Patients after T-Cell-Depleted HLA-Haploidentical Haematopoietic Stem Cell Transplantation Followed by the Reinfusion of iCasp9-Modified Donor T Cells
Source: J Clin Med. 2019 Nov 7;8(11):1904. doi: 10.3390/jcm8111904 (PMC6912839; doi:10.3390/jcm8111904)
Supplement: Supplementary file 1 [file jcm-08-01904-s001.pdf]

Supplementary Table 1. Patient characteristics.

**Patients**	**(n=35)**
**Gender**	
M	22 (63%)
F	13 (37%)
**Age at Transplantation, years (median and range)**	8.48 (1-18)
**Disease**	
ALL	18 (51%)
AML	11 (31%)
MDS	4 (12%)
NHL	2 (6%)
**ALL immunophenotype**	
BCP	17 (95%)
T	1 (5%)
**ALL recurrent molecular lesions**	
t(9;22)(BCR/ABL)	2
t(12;21)(TEL/AML1)	1
t(4;11)(AF4/MLL)	1
3' deletion of MLL	1
t(10;11)(AF10/MLL)	1
**AML recurrent molecular/cytogenetic lesions**	
FLT3-ITD+; DEK-CAN-t(6;9)	1
t(11;12) NUP98-KDM5A	1
FLT3/ITD	1
**Disease status at Transplantation**	
**ALL**	
CR1	5 (28%)
CR2	12 (67%)
CR3	1(5%)
Active disease	-
**AML**	
CR1	6 (55%)
CR2	3 (27%)
CR3	-
Active disease	2 (18%)
**Viral infections/reactivations after HSCT**	
Yes	10 (29%)
No	25 (71%)
**Acute GvHD**	9 (26%)
**Chronic GvHD**	1 (3%)

**Relapse**	3 (9%)
**Conditioning regimens**	
TBI+TT+ Flu	14 (40%)
BU+TT+Flu	8 (23%)
TBI+TT+L-PAM	6 (17%)
Treo+TT+Flu	3 (8%)
TBI+TT+CY	2 (6%)
BU+CY+L-PAM	1 (3%)
BU+FLU+L-PAM	1 (3%)
**Donor characteristics**	
Age (years; range)	40; 21-50
Type of donor	
Mother	16 (46%)
Father	18 (51%)
Sister	1 (3%)
Gender mismatch	16 (46%)
Female Donor -> Male Recipient	10 (28%)
**Cell dose infused, median (range)**	
CD34+ cells x 10^6/kg	19.7 (5.9-33.3)
αβ+ T cells x 10^6/kg	0.04 (0.01-0.09)
γδ+ T cells x 10^6/kg	10.6 (1.26-38.5)
NK cells x 10^6/kg	29.3 (3.35-103.5)
**DLI**	
1 x 10^6	29 (83%)
0,25 x 10^6	1 (3%)
4 x 10^6	5 (14%)

M = male; F = female; ALL = acute lymphoblastic leukemia; AML = acute myeloid leukemia; NHL: Non-Hodgkin Lymphoma; BCP = B-cell precursors; TBI = total body irradiation; TT = thiotepa; Flu = fludarabine; L-PAM = melphalan; BU = busulfan; CY = cyclophosphamide; Treo = treosulfan; CR = complete remission; NK = natural killer;

Supplementary Table 2.

Antigen	Clone	Isotype	Fluorochrome
CD3	SK7	Mouse IgG1	APC-H7
	SK7	Mouse IgG1	FITC
CD16	3G8	Mouse IgG1	PerCP y5.5
CD25	2°3	Mouse IgG1	APC
CD27	M-T271	Mouse IgG1	APC
CD45	2D1	Mouse IgG1	APC H7
	HI30	Mouse IgG1	PE Cy7
CD56	NCAM16.2	Mouse IgG2b	PE
CD57	NK-1	Mouse IgM	FITC
CD62L	DREG-56	Mouse IgG1	APC
CD107a	H4A3	Mouse IgG1	APC
CD122	27302	Mouse IgG1	APC
CD127	h-IL-7R-M21	Mouse IgG1	Alexa Fluor 647
CD158a	HP-3E4	Mouse IgM	FITC
CD158b	CH-L	Mouse IgG2b	FITC
CD158e1	DX9	Mouse IgG1	FITC
CD161	DX12	Mouse IgG1	APC
CXCR3	1C6	Mouse IgG1	APC
CXCR4	12G5	Mouse IgG2a	APC
CX3CR1	2A9-1	Rat IgG2b	APC
NKG2D	1D11	Mouse IgG1	APC
NKG2A	Z199	Mouse IgG1	APC
NKG2C	134591	Mouse IgG1	APC
NKp46	9,00E+02	Mouse IgG1	APC
INF-γ	B27	Mouse IgG1	APC
DNAM-1	DX-11	Mouse IgG1	FITC

Supplementary Table 3: Characteristics of patients used in Figure 2.

**PB CD107a (R)**			**PB CD107a (NR)**		
DISEASE	GVHD	VIRAL INFECTION	DISEASE	GVHD	VIRAL INFECTION
43.5% ALL<br>35% AML<br>17.5% MDS	21.7%	39%	50% ALL<br>30% AML<br>20% NHL	40%	60%

**PB IFN-γ (R)**			**PB IFN-γ (NR)**		
DISEASE	GVHD	VIRAL INFECTION	DISEASE	GVHD	VIRAL INFECTION
45% ALL<br>36% AML<br>9% MDS<br>9% NHL	36%	36%	69.3% ALL<br>30.7% AML	15.4%	23%

**BM CD107a (R)**			**BM CD107a (NR)**		
DISEASE	GVHD	VIRAL INFECTION	DISEASE	GVHD	VIRAL INFECTION
49.5% ALL<br>41.5% AML<br>8% MDS	16.6%	41.6%	33.4% ALL<br>41.6% AML<br>25% MDS	8%	41.6%

**BM IFN-γ (R)**			**BM IFN-γ (NR)**		
DISEASE	GVHD	VIRAL INFECTION	DISEASE	GVHD	VIRAL INFECTION
54.6 % ALL<br>45.4% AML	27%	36%	56.1% ALL<br>28.6% AML<br>14.3% MDS	0%	14.3%

R = responder; NR = non-responder

Supplementary Table 4: Characteristics of patients used in Figure 3.

**PB CD107a (R) – IFN-γ (NR)**			**PB CD107a (NR) – IFN-γ (R)**		
DISEASE	GVHD	VIRAL INFECTION	DISEASE	GVHD	VIRAL INFECTION
62.5% ALL<br>25% AML<br>12.5% MDS	12.5%	37.5%	50% ALL<br>50% AML	25%	50%

**BM CD107a (R) – IFN-γ (NR)**			**BM CD107a (NR) – IFN-γ (R)**		
DISEASE	GVHD	VIRAL INFECTION	DISEASE	GVHD	VIRAL INFECTION
33.4% ALL<br>66.6% AML	0%	33.3%	60% ALL<br>40% AML	20%	40%

R = responder; NR = non-responder

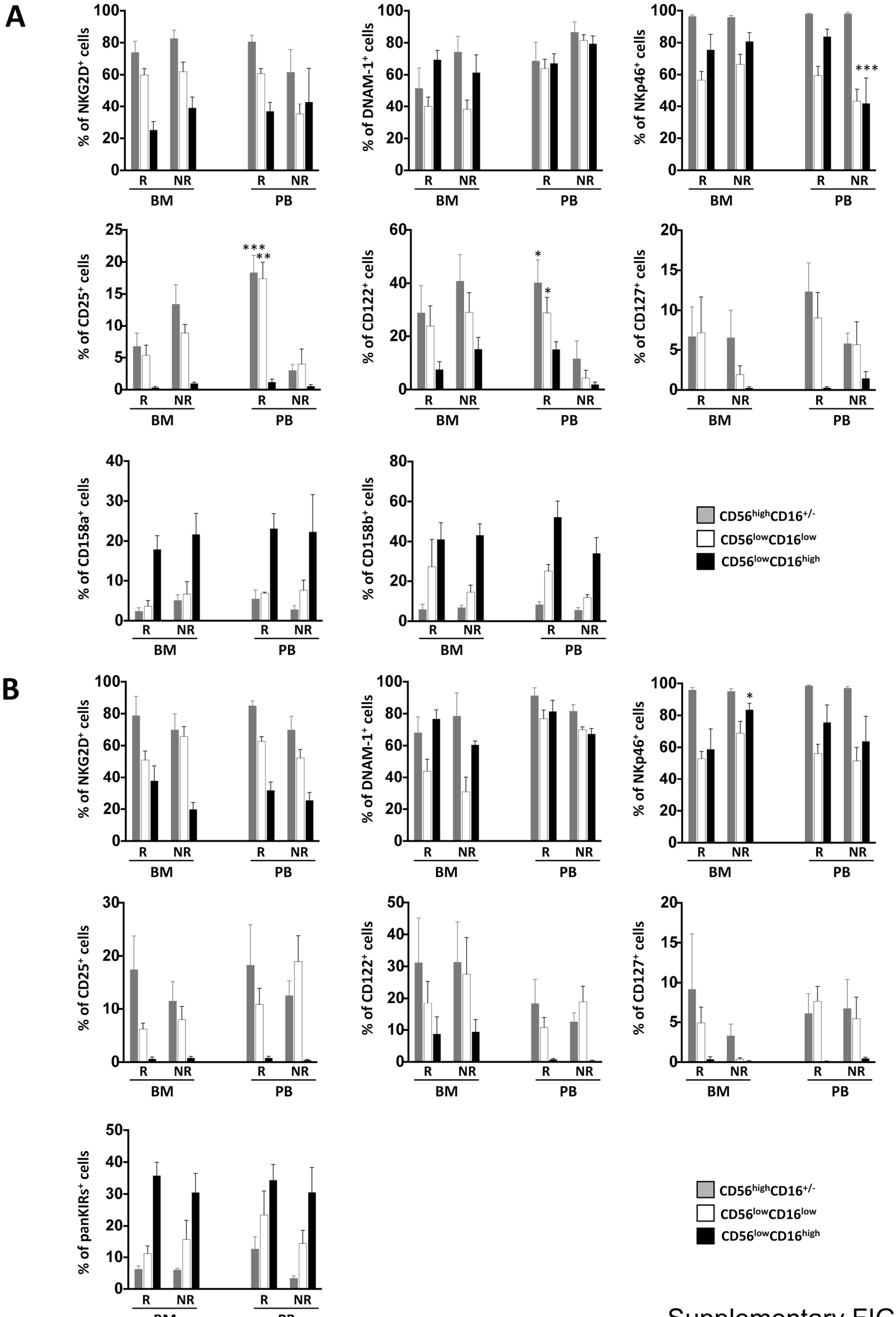

Supplementary FIGURE 1

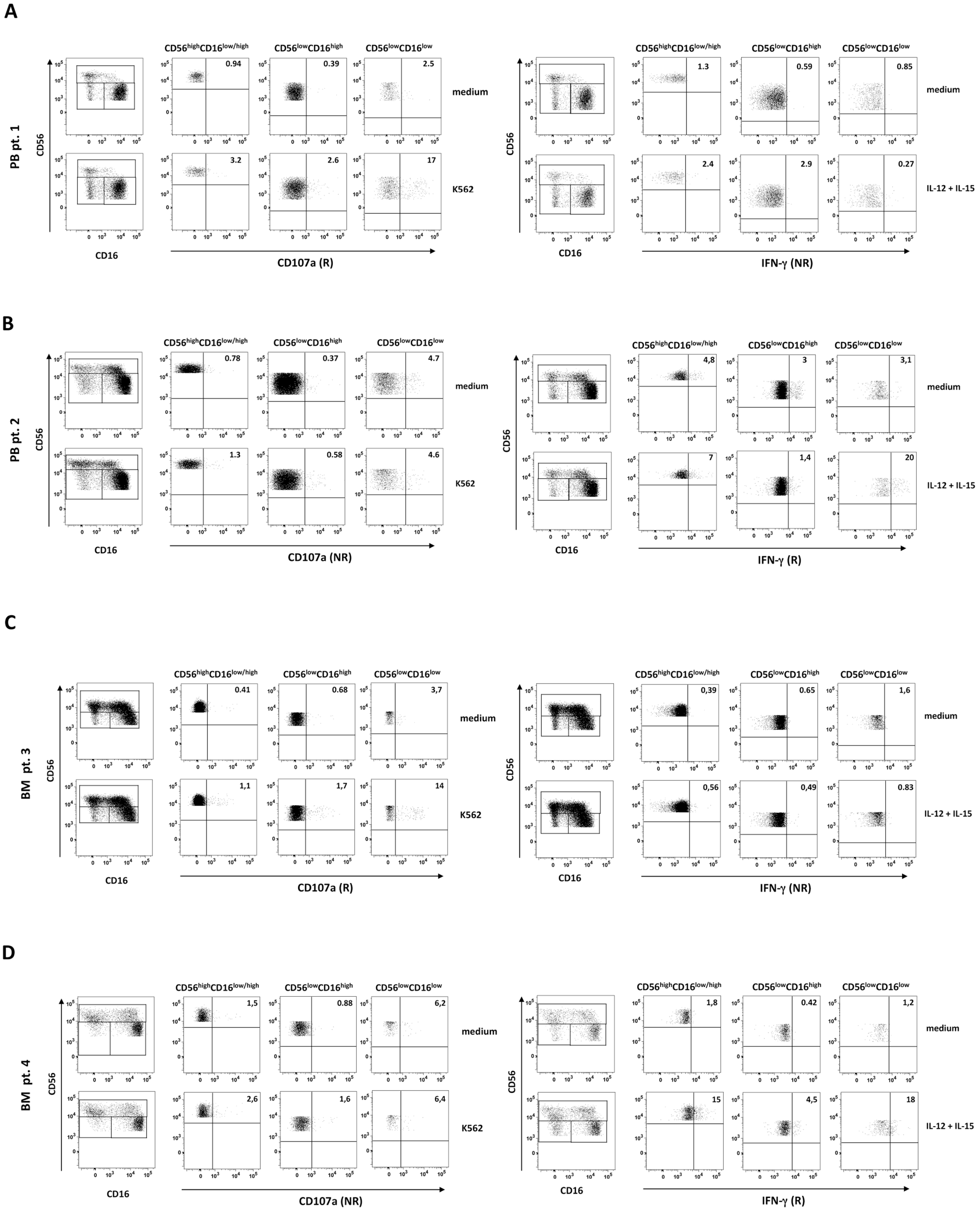

Supplementary FIGURE 2

FUNCTION	$CD56^{low}CD16^{low}$ NK cell subset	
PB and BM CD107a	-	+ (*)
PB and BM IFN-γ	+	-

*** indicate the correlation between degranulation ability of $CD56^{low}CD16^{low}$ NK cells and the frequence of $CD19^{+}CD3^{+}$ cells.**

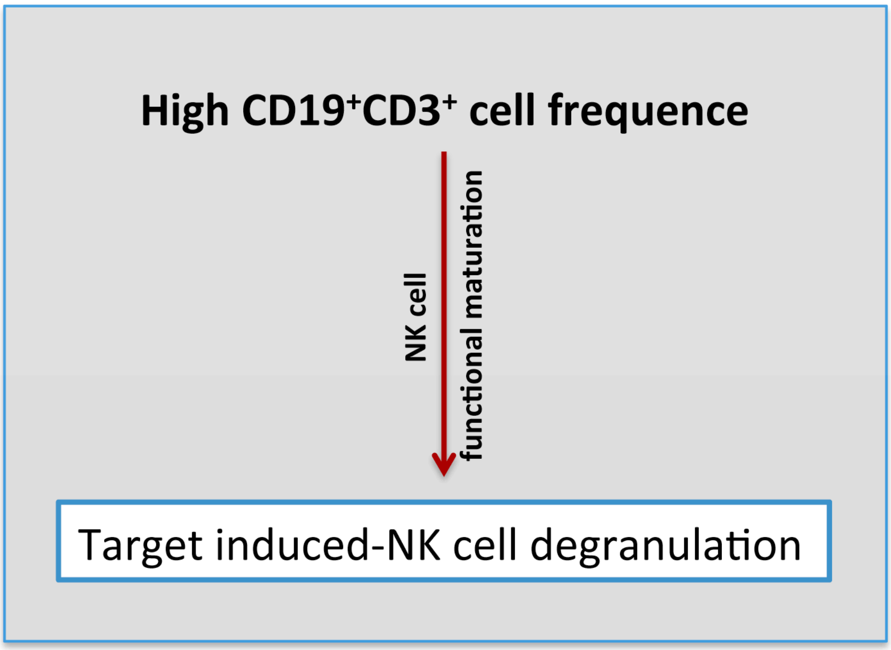

Supplementary FIGURE 3
